# Diversity of *Fusarium* Species Isolated from Symptomatic Plants Belonging to a Wide Range of Agri-Food and Ornamental Crops in Lebanon

**DOI:** 10.3390/jof8090897

**Published:** 2022-08-23

**Authors:** Charlie Abi Saad, Mario Masiello, Wassim Habib, Elvis Gerges, Simona Marianna Sanzani, Antonio Francesco Logrieco, Antonio Moretti, Stefania Somma

**Affiliations:** 1 Institute of Science of Food Production-ISPA, Research National Council—CNR, Via Amendola 122/O, 70126 Bari, Italy; 2 International Center for Advanced Mediterranean Agronomic Studies-CIHEAM Bari, Via Ceglie 9, Valenzano, 70010 Bari, Italy; 3 Centro di Ricerca, Sperimentazione e Formazione in Agricoltura “Basile Caramia”-CRSFA, Via Cisternino 281, Locorotondo, 70010 Bari, Italy; 4 Laboratory of Mycology, Department of Plant Protection, Lebanese Agricultural Research Institute, Fanar 1202, Lebanon; 5 Dipartimento di Scienze del Suolo, della Pianta e degli Alimenti, Università degli Studi di Bari Aldo Moro, Via Amendola 165/A, 70126 Bari, Italy

**Keywords:** Lebanese plants, *Fusarium* species complexes, mycotoxins

## Abstract

Lebanon is a small Mediterranean country with different pedoclimatic conditions that allow the growth of both temperate and tropical plants. Currently, few studies are available on the occurrence and diversity of *Fusarium* species on Lebanese crops. A wide population of *Fusarium* strains was isolated from different symptomatic plants in the last 10 years. In the present investigation, a set of 134 representative strains were molecularly identified by sequencing the translation elongation factor, used in *Fusarium* as a barcoding gene. Great variability was observed, since the strains were grouped into nine different *Fusarium* Species Complexes (SCs). *Fusarium oxysporum* SC and *Fusarium solani* SC were the most frequent (53% and 24%, respectively). Members of important mycotoxigenic SCs were also detected: *F. fujikuroi* SC (7%), *F. sambucinum* SC (5%), *F. incarnatum-equiseti* SC (3%), and *F. tricinctum* SC (4%). Two strains belonging to *F. lateritium* SC, a single strain belonging to *F. burgessii* SC, and a single strain belonging to *F. redolens* SC were also detected. This paper reports, for the first time, the occurrence of several *Fusarium* species on Lebanese host plants. The clear picture of the *Fusarium* species distribution provided in this study can pose a basis for both a better understanding of the potential phytopathological and toxicological risks and planning future *Fusarium* management strategies in Lebanon.

## 1. Introduction

Lebanon is one of the smallest countries in the Mediterranean basin. Although it has a limited agricultural area of about 650,000 ha [1], the variety of agricultural land and climatic conditions enables farmers to grow a plethora of plants, including both temperate crops (e.g., grape, olive tree, and solanaceous plants) and tropical crops (e.g., banana and avocado). The territory is quite varied, ranging from the flat coastal area to the mountain chain in the center of the country, where large areas are occupied by cedar and fir woods. Generally, tobacco and figs are grown in the southern part of Lebanon, citrus fruits and banana along the coast, olives and apples in different parts of the Mount Lebanon governorate, and, in the north, fruits, vegetables, and cereals in the fertile Bekaa Valley. Lebanon exports part of its production to other Middle Eastern countries and Arab Gulf states [1].

Several factors, including pedo-climatic conditions, poor agricultural and storage practices, and the weakness of the food safety management system, can simultaneously lead to contamination of Lebanese agri-food commodities by toxigenic fungi and their mycotoxins [2]. This represents both a phytopathological issue and a serious risk to consumers’ health. In recent decades, the socio-economic disorder due to the long civil war and the political instability have not allowed the improvement of the weak national food safety system. Only in 2015, for the first time, was the Food Safety Lebanese Commission established, although the new political instability is compromising its activities [2].

Under Lebanese climatic conditions, all crops could be potentially colonized by the most important mycotoxigenic fungal species belonging to the *Alternaria*, *Aspergillus*, *Fusarium*, and *Penicillium* genera. However, to date, few studies are available in the literature on the biodiversity of phytopathogenic fungi and the accumulation of mycotoxins in Lebanese crops and agri-food commodities. Recently, thorough surveys and phylogenetic studies were carried out by Habib et al. [3,4,5] to evaluate the distribution and the toxicological risk of *Alternaria* and *Penicillium* species on wheat and tomato at preharvest, and apple and table grape at postharvest. On the other hand, El Darra et al. [6] evaluated the occurrence of mycotoxins in spices and herbs commercialized in Lebanon, showing the high level of mycotoxins in spices consumed in Lebanon. A few studies have also reported the occurrence of *Aspergillus* and *Penicillium* species and their mycotoxins on wheat, grape, and other commodities [7,8,9,10,11]. On the contrary, very little information is available on the occurrence and diversity of *Fusarium* species on Lebanese crops. Such information includes the report by Ordonez et al. [12] on the occurrence of *Fusarium oxysporum* f.sp. *cubense* on banana, and the reports on *Fusarium redolens* and *F. oxysporum* isolated from chickpea [13,14].

The *Fusarium* genus includes several phytopathogenic species, such as *F. graminearum* and *F. oxysporum*, which are two causal agents of the most important plant diseases worldwide [15]. Nowadays, based on genetic analyses, taxonomists have grouped *Fusarium* species into 23 well-separated phylogenetic species complexes [16,17]. Worldwide, the most important phytopathogenic *Fusarium* species belong to the *Fusarium sambucinum* species complex (FSAMSC), *Fusarium fujikuroi* species complex (FFSC), *Fusarium solani* species complex (FSSC), and *Fusarium oxysporum* species complex (FOSC). To a lesser extent, members of the *Fusarium incarnatum-equiseti* species complex (FIESC), regarded as moderate pathogens, have been isolated together with other *Fusarium* species from different diseased plants, such as cereals, sugarcane, and date palm [18,19,20,21].

In particular, *Fusarium* species included in the FSAMSC and FFSC represent both a phytopathological issue and a serious toxicological risk for humans and animals, although these two aspects are rarely related. Indeed, almost all 78 and 56 species included in the FSAMSC and FFSC, respectively, can simultaneously produce multiple mycotoxins [16]. In particular, the most important mycotoxins produced by FSAMSC are type A and type B trichothecenes, such as HT-2 and T-2 toxins, nivalenol, deoxynivalenol (DON) and their acetylated forms, and zearalenone [22]. The most dangerous mycotoxins produced by FFSC are fumonisins B (FBs), occurring worldwide, primarily in maize, and especially in warm climates [23]. The most important among FBs is FB_1_, classified by IARC as a possible carcinogen to humans [24]. Together with FBs, members of the FFSC are also able to produce so-called emerging mycotoxins, such as beauvericin, enniatins, fusaric acid, and moniliformin [25]. These emerging mycotoxins can be synthesized by species belonging to different species complexes, such as the FOSC, FIESC, and *Fusarium tricinctum* species complex (FTSC) [16]. BEA and ENNs B are reported to possess phytotoxic properties, acting as virulence factors in tomato and potato plant infections [26,27].

Members of the FOSC and FSSC are of great concern, because they are the most important causal agents of vascular wilt and root rot in diverse crops. The most important diseases caused by *F. oxysporum* include Fusarium wilt of banana, common bean, cotton, lettuce, and tomato. However, it must be highlighted that the FOSC also includes many populations of strains that are not pathogenic, with the pathogenic ones usually belonging to host-specific formae speciales. The FSSC includes causal agents of soybean sudden death syndrome, bean root rot, and multiple vegetable diseases [28,29]. In addition, some members of the FSSC can be involved in human and animal infections.

Since the *Fusarium* species distribution is widely influenced by environmental conditions, and several species showing different mycotoxin profiles can be largely isolated together from symptomatic plants, a correct identification of *Fusarium* species can allow for a clear picture of the potential phytopathological and toxicological risks. From this perspective, the aim of this study was to identify a wide set of *Fusarium* strains isolated from different agriculturally important crops in Lebanon to obtain, for the first time, a large amount of information on the *Fusarium* species diversity in this country.

## 2. Materials and Methods

### 2.1. Sampling 

**Figure 1:**
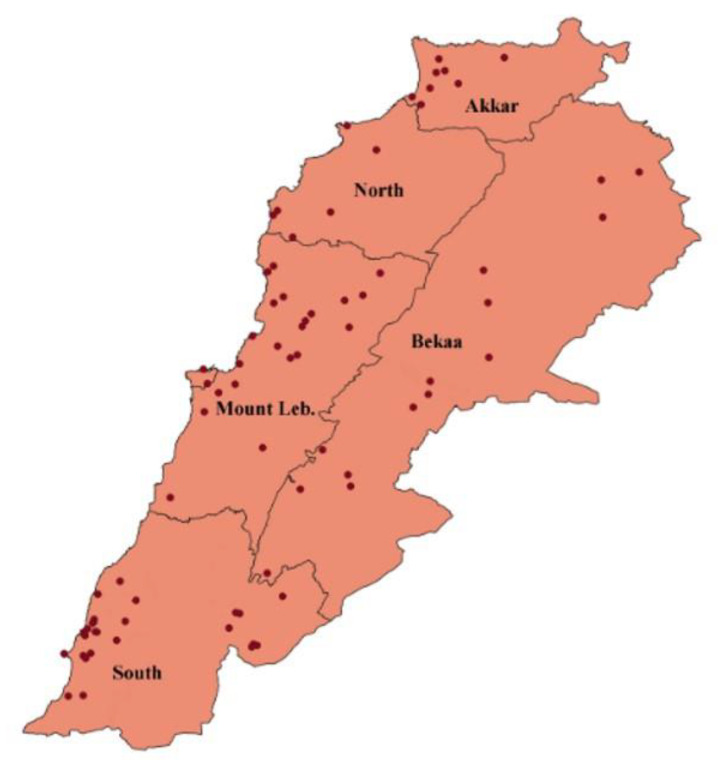
Sites on the Lebanese map of host plant sampling.

During a 10-year period, from 2012 to 2021, and within the service of the plant disease diagnostic clinic provided by the Lebanese Agricultural Research Institute (LARI), symptomatic plant tissues were brought by private companies, public institutions, and farmers from all over Lebanon (Figure 1). *Fusarium* colonies, isolated from different host plants, including agri-food plants, ornamental plants, and forest trees, were selected to obtain a collection of 134 *Fusarium* strains (Table 1). 

### 2.2. Morphological Identification of Fusarium Strains 

For fungal isolation, after surface disinfection with 70% ethanol or 2% sodium hypochlorite solution and washing twice with sterile distilled water, small portions (about 5 mm × 5 mm) taken from the margin of the infected areas were transferred to potato dextrose agar plates (PDA, Merck, Darmstadt, Germany) and incubated for 5–7 days at 25 °C until colony development. 

To obtain pure Fusarium strains from the developing fungal colonies, a mass of conidia was resuspended in sterile distilled water and scattered at low density on 90 mm water agar (WA) Petri dishes. After 18–24 h of incubation at 25 °C in the darkness, germinated conidia were singularly transferred to 60 mm PDA plates using a dissecting microscope.

After 5–7 days of incubation at 25 °C under a 12 h photoperiod, Fusarium strains cultured on PDA and Spezieller Nahrstoffarmer agar (SNA) media were morphologically identified according to Nelson et al. and Leslie and Summerell [30,31].

### 2.3. Molecular Identification of the Fusarium Species by Translation Elongation Factor 1-α Sequencing

#### 2.3.1. DNA Extraction 

For each monosporic strain, 5 mycelium plugs were collected from the margin of actively growing colonies on PDA and transferred to a sterilized cellophane disk overlaid on PDA 90 mm plates. After incubation at 25 °C for 2–3 days, the mycelia were collected into 2 mL tubes, frozen, and lyophilized. The DNA extraction was carried out on 15 mg of powdered lyophilized mycelium by using a Wizard Magnetic DNA Purification System for Food kit (Promega Corporation, Madison, WI, USA) according to the manufacturer’s protocol. The genomic DNA quantity and integrity were checked on 0.8% agarose gel in 1X Tris-Acetate-EDTA (TAE) using a standard 1 kb DNA Ladder (ThermoFisher Scientific, Carlsbad, CA, USA) and a Nanodrop spectrophotometer (ThermoFisher Scientific).

#### 2.3.2. Translation Elongation Factor 1-α (TEF) Amplification and Sequencing Analysis

For each Fusarium strain, the informative fragment of the Translation elongation factor 1-α gene was amplified and sequenced using the primer pair EF-1 and EF-2 [32]. PCR reactions were carried out in a 15 μL final volume containing 15 ng of genomic DNA, 300 nM of each primer, 200 nM dNTPs, 1× PCR buffer, and 0.6 U of Hot Start Taq DNA Polymerase (Fisher Molecular Biology, Trevose, PA, USA). The amplifications were carried out in a Mastercycler EP Gradient thermal cycler (Eppendorf, Hamburg, Germany) under the following conditions: initial denaturation of 2 min at 95 °C, followed by 35 cycles of 50 s at 95 °C, 50 s at 59 °C, 1 min at 72 °C, and a final extension of 7 min at 72 °C.

The PCR products, stained with GelRed^®^ (Biotium Inc., Fremont, CA, USA), were checked after electrophoretic separation on 1.5% agarose gel in 1× TAE buffer under UV light by comparison with a 100 bp DNA Ladder (ThermoFisher Scientific). Before sequencing, the PCR products were purified with an enzymatic EXO/FastAP mixture (ExonucleaseI and FastAP thermosensitive alkaline phosphatase, ThermoFisher Scientific). Sequence reactions were performed for both strands using a BigDye Terminator v3.1 Cycle Sequencing Ready Reaction Kit (Applied Biosystems, Foster City, CA, USA) according to the manufacturer’s recommendations. The labeled products were purified by filtration through Sephadex G-50 (5%) (Sigma-Aldrich, Saint Louis, MO, USA) and analyzed using an ABI PRISM 3730 Genetic Analyzer (ThermoFisher Scientific). The FASTA sequences obtained were analyzed and assembled using BioNumerics v. 5.1 software (Applied Maths, Kortrijk, Belgium). 

For molecular identification at the species level, the obtained sequences were compared with the sequences available in the NCBI Database through the BLASTN program. To further solve the phylogenetic relationships between and within the Fusarium species detected, the sequences obtained in this study joined to 23 sequences of Fusarium reference strains and to the sequence of Ilyonectria crassa CBS 158.31 strain, used as an outgroup, were aligned by using the MUSCLE algorithm [33]. Phylogenetic relationships were analyzed using the maximum likelihood method with MEGA software version 7 [34]. Bootstrap analyses [35] were conducted to determine the confidence of internal nodes using a heuristic search with 1000 replicates, removing gaps.

## 3. Results 

In Table 1, 134 Fusarium strains, isolated from 38 different host plants, are listed. In particular, the strains were isolated from different plant parts, such as the roots, crown area, stem, and fruits, showing symptoms of fungal diseases, such as wilting, vascular browning, yellowing, necrotic or chlorotic leaf spots, crown rot, and also decline or death. For each Fusarium strain, the geographical and host plant origin, organ of isolation, and symptoms observed are detailed. The majority of the strains were isolated from plants grown in the South and Bekaa regions (45 and 42 strains, respectively), followed by Mount Lebanon (25 strains) and Akkar (17 strains) regions. Four Fusarium strains were also collected in the North, and a single strain was obtained from an ornamental plant grown in Beirut city (Table 1).

**Figure 2:**
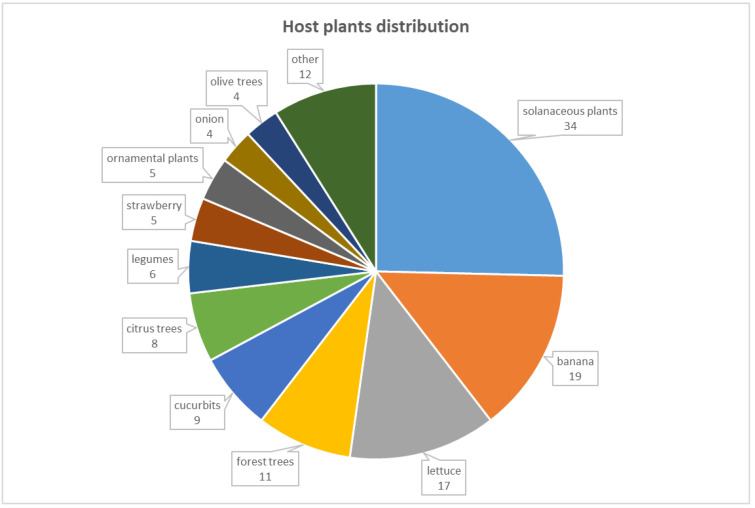
Lebanese host plants from which *Fusarium* strains (shown for each group of plants in the graph) were isolated: solanaceous plants (potato 20, tomato 8, eggplant 4, tobacco 1, and hot pepper 1); banana, lettuce, citrus trees (citrus 2, lemon 4, grapefruit 1, and mandarin 1), legumes (bean 2, pea 3, and faba bean 1), cucurbits (melon 4, watermelon 3, and cucumber 2), strawberry, forest trees (cedar 5, abies 1, hornbeam 1, pine 3, and walnut tree 1), ornamental plants (ivy 1, poinsettia 2, Jacaranda 1, and roses 1), olive tree, and onion. To a lesser extent, *Fusarium* strains were isolated from other host plants (apple 3, avocado 3, kiwi 1, cauliflower 1, basil 1, parsley 1, and paspalum grass 1).

Host plants affected by Fusarium species were grouped considering their botanical and commodity classifications, as shown in Figure 2. Most of the Fusarium strains were isolated from solanaceous plants (34 out of 134), banana (19 out of 134) and lettuce (17 out of 134), together representing half of the Fusarium strains isolated from symptomatic plants. In detail, 20 strains were isolated from potato, 8 strains from tomato, 4 strains from eggplant, and a single Fusarium strain from tobacco and hot pepper, respectively (Table 1). To a lesser extent, Fusarium strains were isolated from forest tree (11 strains), cucurbits (9 strains), citrus trees (8 strains), legume (6 strains), strawberry (5 strains), ornamental plants (5 strains), and onion and olive tree (4 strains, respectively). As reported in Table 1, 12 Fusarium strains were also isolated from other host plants, including apple (3 strains), avocado, parsley, and Paspalum grass (2 strains from each), and basil, cauliflower, and kiwi (a single strain from each). The distributions of Fusarium species in the different geographical regions are reported in Table 2. The single strain isolated in Beirut, from the ornamental Jacaranda tree, was identified as a member of the FSSC. In the North Lebanon region, four Fusarium strains were isolated from tomato, pea, and strawberry, and identified as FOSC. A large number of Fusarium strains were isolated from plants grown in the Akkar, Bekaa, Mount Lebanon, and South Lebanon regions. In all of these regions, the FOSC and FSSC were the most dominant SCs, with values ranging between 43.8 (Akkar) and 59.5% (Bekaa), and between 12.5 (Akkar) and 31% (Bekaa) (Table 2). In addition to the FOSC and FSSC strains, in the Southern region, great variability among Fusarium species isolated was observed, with high frequency of the FFSC (13.3%), followed by the FTSC and FLSC (4.4%), and, to a lesser extent, by FIESC, FSAMSC, and FBSC (2.2%). Fusarium redolens strains were isolated only from plants grown in the Mount Lebanon region, with a value of 4%. In this region, strains belonging to the FTSC (8%), FIESC (8%), and FSAMSC (4%) were also isolated. In the Bekaa region, strains belonging to the FSAMSC and FFSC, with values of 4.8%, were isolated. In the Akkar region, 6.3% of the strains belonged to the FIESC, and the same value of 12.5% of strains was recorded for each of the following SCs: FSAMSC, FFSC, and FTSC (Table 2).

**Figure 3:**
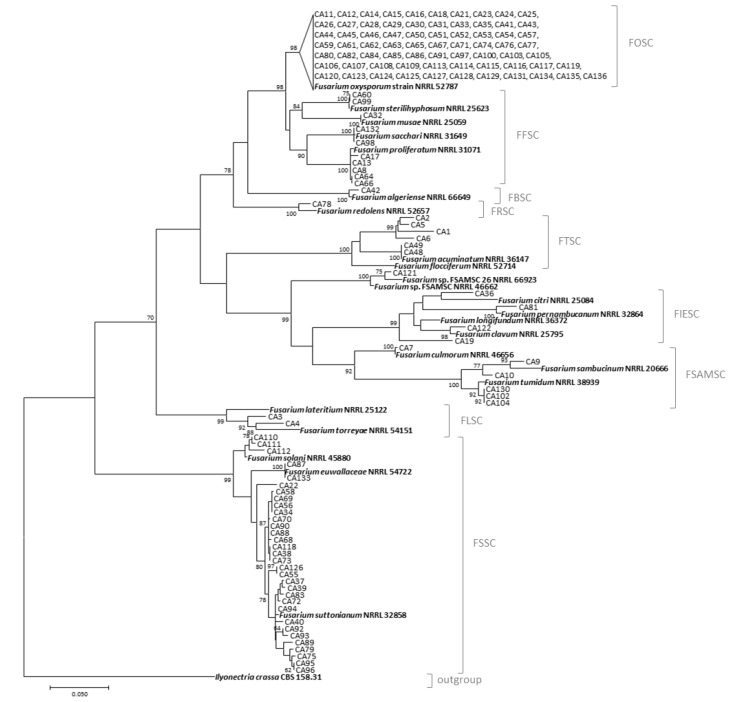
Phylogenetic tree built with the TEF sequences of 134 Lebanese *Fusarium* strains and 23 species reference strains. The phylogenetic tree was generated by the maximum likelihood method; bootstrap values > 70 with 1000 replicates are shown near the branches. Fusarium species complexes are also shown in the clustering.

The phylogenetic analysis of the TEF sequences of 134 Lebanese *Fusarium* strains, 23 *Fusarium* reference sequences, and 1 sequence of *Ilyonectria crassa* used as an outgroup taxon resulted in the tree shown in Figure 3. The phylogenetic tree was resolved in 11 well-separated clades, supported by high bootstrap values (over 98%). A large set of 71 strains clustered together and with the reference strain NRRL 52787, belonging to the *F. oxysporum* species complex (FOSC). Ten strains were identified as belonging to the *F. fujikuroi* species complex (FFSC); in particular, two strains were identified as *F. steriliphosum*, one strain as *F. musae*, two as *F. sacchari,* and five strains showed high similarity to the *F. proliferatum* reference strain. Another 20 strains clustered in six well-supported clades, corresponding to the *F. burgessii* species complex (FBSC), *F. redolens* species complex (FRSC), *F. tricinctum* species complex (FTSC), *F. incarnatum-equiseti* species complex (FIESC), *F. sambucinum* species complex (FSAMSC), and *F. lateritium* species complex (FLSC). The FBSC and FRSC were both represented by only one strain and the corresponding species reference strain (*F. algeriense* and *F. redolens*, respectively). In detail, six strains belonged to the FTSC, among which two strains showed homology with the *F. acuminatum* NRRL 36147 reference strain. Among four FIESC strains, one strain showed high similarity to the *F. citri* reference strain, one strain to *F. pernambucanum*, and one strain to *F. clavum*. The FSAMSC clade included one strain identified as *F. culmorum*, two strains clustered with the *F. sambucinum* reference strain, and three strains sharing high homology with the reference strain *F. tumidum* NRRL 38939. One strain showed similarity to the reference strains NRRL 66923 and NRRL 46662, belonging to the FSAMSC, but this well-supported clade (100 bootstrap value) did not group with the other species of the FSAMSC described previously. Two strains were identified as belonging to the FLSC clade. Another large clade grouping 32 Lebanese strains was the *Fusarium solani* species complex (FSSC). Most of these strains showed similarity to the *F. suttonianum* reference strain NRRL 32858; two strains were identified as *F. euwallaceae*, and three strains clustered with *F. solani* NRRL 45880.

**Figure 4:**
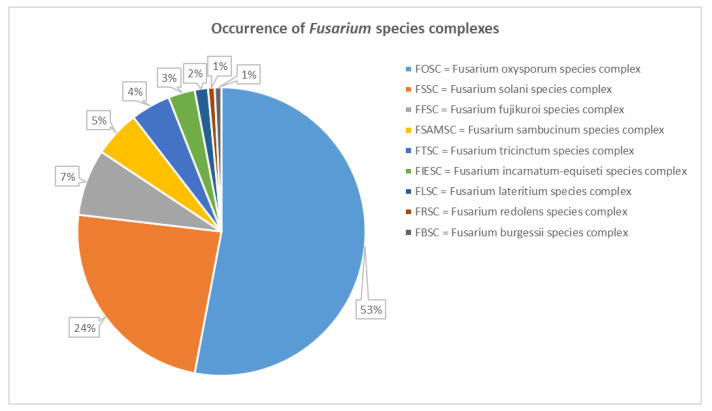
Occurrence of *Fusarium* species complexes, molecularly identified by TEF sequences, in Lebanese plants.

The occurrence of the different *Fusarium* species complexes identified through the phylogenetic analysis of TEF sequences in Lebanese plants is shown in Figure 4. The FOSC was the most widespread species complex in Lebanon, representing 53% of the overall 134 collected strains, followed by the FSSC, with a percentage of 24%. Other species complexes occurring in different plants at 7, 5, and 4% are the FFSC, FSAMSC, and FTSC, respectively. To a lesser extent, strains belonging to the FIESC (3%) and FLSC (2%) were identified. Finally, one strain of *F. redolens* from parsley crown and one strain of *F. algeriense* from potato crown and roots were isolated.

## 4. Discussion

A wide population of *Fusarium* strains infecting plants all over Lebanon in the last 10 years has been isolated and identified to achieve, for the first time, extended information regarding *Fusarium* diversity occurring on Lebanese crops. Furthermore, since coniferous forests are a primary constituent of the Lebanese landscape, the cedar of Lebanon is included in this study, being the national emblem of the country. Indeed, monitoring activities to assess *Fusarium*’s occurrence in a wide range of plants in Lebanon were lacking, as well as updated information regarding the risk related to *Fusarium* mycotoxins. Plants showing mostly symptoms of decline and withering, were collected from different areas of the country and belonged to a wide range of categories, ranging from agri-food to ornamental plants, including herbaceous plants and forest trees. The most represented commodities in the *Fusarium* host collection were potato, banana, and lettuce. In particular, potato is one of the primary crops grown in Lebanon for local consumption, processing, and export, representing more than 45% of the total area assigned to vegetable crops [1], and banana is one of the main crops intended for both local consumption and export, mostly in South Lebanon. Therefore, it is highly worrisome that such important crops for Lebanese agriculture showed such high *Fusarium* occurrence.

The molecular identification, based on TEF sequencing, allowed distinguishing several *Fusarium* species that, according to the contemporary *Fusarium* species concept [16], have been assigned to given *Fusarium* species complexes. A total of nine different species complexes occurred among the *Fusarium* strains herein, demonstrating that a wide biodiversity of *Fusarium* is detectable in Lebanon.

The most represented species complex (53%) was the FOSC, whose members were isolated from almost all of the plants considered in this study. Members of this complex are well-known causal agents of a wide range of diseases, including Panama disease on banana, and Fusarium wilt and root and crown rot on legumes, lettuce, solanaceous plants, and cucurbits [36]. In our study, the plants from which *F. oxysporum* strains were recovered are commonly known in the literature as host crops of this ubiquitous species. Furthermore, these strains were isolated from the crowns and roots of plants showing yellowing, decline, wilt, and rot, which are symptoms of diseases caused by *F. oxysporum* (Table 1). Besides the economic damage caused by their pathogenicity to plants, FOSC members are able to produce toxic metabolites, such as beauvericin (BEA), a phytotoxic compound contributing to pathogenicity in tomato; fusaric acid (FA), known for its ability to induce wilt symptoms in several plants; and enniatins (ENNs). In addition, each of these metabolites has been proven to be toxic to human and animal cell lines or animals fed with contaminated feed [23]. These results encourage the consideration of prevention measures to be adopted by studying these pathogens and control methods aimed to prevent heavy deterioration and yield losses in Lebanese fields.

The second-most-occurring species complex in Lebanon in the present study was the FSSC (24%). Similar to FOSC members, members of this complex are known to include species causing root and crown rot. Consistently, Lebanese FSSC strains were isolated from several different host plants showing browning, necrosis, decline, and rot. Although the FSSC does not contribute to mycotoxin contamination, this complex includes more than 80 species sharing very high genetic variability and is considered among the most relevant pathogenic group of species in the genus.

Both the FOSC and FSSC are present worldwide, but *F. oxysporum* has been previously reported in Lebanon only associated with Panama disease in banana [12] and in chickpea [13]. In the present investigation, nine strains belonging to FFSC were isolated from banana, potato, lettuce, strawberry, olive tree, and the cedar of Lebanon. Among the four strains isolated from banana tree, the only one from fruits was identified as *F. musae*, confirming the strong association of this species with banana fruits, whereas the other strains isolated from banana stems were *F. sacchari*, *F. steriliphosum,* and *F. proliferatum*. Only one FFSC strain from potato tubers was identified as *F. proliferatum*, similar to the other strains from strawberry, olive tree, and cedar. Although the number of occurring strains is limited, the presence of *F. proliferatum* is of great concern for its ability to produce different mycotoxins, mainly FBs, which have been associated with a wide number of animal diseases and are classified by IARC as potentially carcinogenic [37].

Two other species complexes identified in Lebanese strains of great importance for the presence of toxigenic species are the FSAMSC and FIESC. However, except for one *F. culmorum* strain from eggplant, which produces DON, the species identified within the SAMSC were mainly *F. sambucinum* and *F. tumidum*, both producing trichothecene type A mycotoxins. Conversely, FIESC species, detected in citrus trees, melon fruits, and ornamental plants are known to produce multiple mycotoxins, including type A and B trichothecenes, zearalenone, BEA, and ENNs.

On the other hand, 4% of the studied *Fusarium* strains belonged to the FTSC, characterized by widespread species, but producing only BEA and ENNs. These strains were recovered only from citrus trees and from apple fruits. Similarly, *F. avenaceum* was reported as a postharvest pathogen of apples in Italy [38] and the Netherlands [39].

Furthermore, sporadic strains belonging to the FLSC (two strains from lemon), FRSC (a single strain from parsley), and FBSC (a single *F. algeriense* strain from potato root and crown) were identified. These *Fusarium* species can produce mycotoxins, such as BEA, ENNs, FA, and MON. *Fusarium algeriense*, is a recent species identified only on durum wheat in Algeria, as far as we are aware [40]. On the contrary, *F. redolens,* causing wilt decline and death in the parsley plants investigated in our study, has been previously reported in Lebanon on chickpea [14].

## 5. Conclusions

Lebanon produces several important agricultural commodities, due to the climatic conditions favorable to agriculture, that are even exported to several other countries. Since investigations into the occurrence of *Fusarium* in Lebanon are limited, an extended investigation of *Fusarium* occurrence was conducted, focused on a widespread monitoring of several Lebanese plants of agri-food and ornamental interest. The *Fusarium* genus was proven to be widely spread, with very high biodiversity, including several species that could represent a risk to consumers due to their potential mycotoxin production. The collected strains were distributed in nine different species complexes, with the FOSC and FSSC being the most present. Other important species complexes of great concern, such as the FFSC, FSAMSC, FIESC, and FTSC, were also identified. To a lesser extent, a few strains belonging to the FLSC, FBSC, and FRSC were detected. Most of the species detected in the present investigation are reported for the first time in Lebanon, since few investigations focused on the *Fusarium* genus in this country. The wide distribution and diversity of *Fusarium* around the country suggest a lack of effective disease management, correct identification, and good agricultural practices to prevent any new occurrence and dispersal of pathogens between fields. In addition, this investigation shows that a policy to evaluate the regulation of *Fusarium* mycotoxins in Lebanon is needed and must be undertaken.

## Figures and Tables

**Table 1:** List of *Fusarium* strains isolated from Lebanese plants. The geographical origin, host plant, observed symptoms, part of the plants from which the strains were isolated, and the identified *Fusarium* species complex are reported for each *Fusarium* strain.

Strain Code	Lebanese Region	Host Plant	Organ	Symptoms	Fusarium Species Complex
CA135	Bekaa	Eggplant	Crown area	Wilt, decline, death	FOSC
CA136	Bekaa	Eggplant	Crown area	Wilt, decline, death	FOSC
CA53	Akkar	Eggplant	Roots, crown	Vascular browning, wilt, decline	FOSC
CA7	Bekaa	Eggplant	Stem, petioles	Wilt, decline	FSAMSC
CA42	South	Potato	Roots, crown	Internal browning	FBSC
CA13	Akkar	Potato	Seed tubers	Dry rot	FFSC
CA25	Akkar	Potato	Roots, crown	Wilt, decline	FOSC
CA26	Akkar	Potato	Roots, crown	Wilt, decline	FOSC
CA27	Akkar	Potato	Roots, crown	Wilt, decline	FOSC
CA28	Akkar	Potato	Roots, crown	Wilt, decline	FOSC
CA29	Akkar	Potato	Roots, crown	Wilt, decline	FOSC
CA30	Bekaa	Potato	Roots, crown	Wilt, death	FOSC
CA41	South	Potato	Roots, crown	Internal browning	FOSC
CA120	Bekaa	Potato	Stem	Necrotic leaves, necrotic spots on stem	FOSC
CA9	Akkar	Potato	Roots, tubers	Tuber seeds unusual growth	FSAMSC
CA130	Bekaa	Potato	Tubers	Spoiled tubers, white mycelium on tubers	FSAMSC
CA37	South	Potato	Roots, crown	Internal browning	FSSC
CA38	South	Potato	Roots, crown	Internal browning	FSSC
CA39	South	Potato	Roots, crown	Internal browning	FSSC
CA40	South	Potato	Roots, crown	Internal browning	FSSC
CA22	Akkar	Potato	Seed tubers	Dry rot	FSSC
CA114	Bekaa	Potato	Roots, crown	Decline, black lesions on roots	FOSC
CA10	Akkar	Potato	Roots, tubers	Tuber seeds unusual growth	FSAMSC
CA102	Akkar	Potato	Tuber	Necrotic spots and lesions	FSAMSC
CA55	Akkar	Tobacco	Collar area	Wilt, yellowing, chlorotic leaves, death	FSSC
CA61	Mount Leb.	Tomato	Collar area	Poor growth, weakness	FOSC
CA134	North	Tomato	Roots	Wilt, decline, death	FOSC
CA67	Mount Leb.	Tomato	Roots, crown	Wilt, internal browning	FOSC
CA11	Bekaa	Tomato	Roots, stem	Leaf chlorosis, necrosis, wilt	FOSC
CA14	Mount Leb.	Tomato	Roots, stem	General decline, wilt	FOSC
CA24	Mount Leb.	Tomato	Roots, stem	Necrosis on crown area, wilt	FOSC
CA47	North	Tomato	Stem	Vascular browning, wilt, decline	FOSC
CA88	Bekaa	Tomato	Roots	Corky roots	FSSC
CA54	Akkar	Hot pepper	Roots, crown	Vascular browning, wilt, decline	FOSC
CA32	South	Banana	Fruits	Fruit tip and crown necrosis	FFSC
CA132	South	Banana	Pseudo stem	Decline, yellowing	FFSC
CA105	South	Banana	Crown area	Yellowing, decline, death	FOSC
CA106	South	Banana	Crown area	Yellowing, decline, death	FOSC
CA46	South	Banana	Crown, stem	Wilting, yellowing	FOSC
CA127	South	Banana	Pseudo stem	Leaf yellowing, pseudo stem cracking, internal browning	FOSC
CA128	South	Banana	Pseudo stem	Leaf yellowing, pseudo stem cracking, internal browning	FOSC
CA129	South	Banana	Pseudo stem	Leaf yellowing, pseudo stem cracking, internal browning	FOSC
CA31	South	Banana	Roots, crown	Wilt, internal browning	FOSC
CA119	South	Banana	Stem	Internal discoloration, decline, death	FOSC
CA98	South	Banana	Stem	Yellowing, decline, death	FFSC
CA99	South	Banana	Stem	Yellowing, decline, death	FFSC
CA100	South	Banana	Crown area	Yellowing, decline, death	FOSC
CA103	South	Banana	Crown area	Yellowing, decline, death	FOSC
CA97	South	Banana	Stem	Yellowing, decline, death	FOSC
CA92	South	Banana	Crown area	Internal browning and necrosis	FSSC
CA93	South	Banana	Crown area	Internal browning and necrosis	FSSC
CA95	South	Banana	Crown area	Yellowing, decline, death	FSSC
CA96	South	Banana	Crown area	Yellowing, decline, death	FSSC
CA60	Bekaa	Lettuce	Collar area	Wilt, decline, death	FFSC
CA52	Bekaa	Lettuce	Collar area	Dry rot, wilt, chlorotic leaves	FOSC
CA57	Bekaa	Lettuce	Collar area	Yellowing, wilting, dry rot	FOSC
CA74	Bekaa	Lettuce	Crown area	Yellowing, wilting, dry rot	FOSC
CA77	Bekaa	Lettuce	Crown area	Yellowing, stunting, death	FOSC
CA85	Bekaa	Lettuce	Crown area	Wilt, decline, death	FOSC
CA108	Bekaa	Lettuce	Crown area	Dry rot	FOSC
CA115	Bekaa	Lettuce	Crown area	Wilt, decline, death	FOSC
CA116	Bekaa	Lettuce	Crown area	Wilt, decline, death	FOSC
CA125	Bekaa	Lettuce	Crown area	Decline, death, vascular discoloration	FOSC
CA33	Bekaa	Lettuce	Roots, crown	Root rot	FOSC
CA82	Bekaa	Lettuce	Roots, crown	Wilt, decline, death	FOSC
CA107	Bekaa	Lettuce	Roots, crown	Decline, internal browning	FOSC
CA56	Bekaa	Lettuce	Collar area	yellowing, wilting, dry rot	FSSC
CA58	Bekaa	Lettuce	Collar area	yellowing, wilting, dry rot	FSSC
CA79	South	Lettuce	Crown area	Wilt, decline, death	FSSC
CA118	Bekaa	Lettuce	Crown area	Black necrotic spots on collar area	FSSC
CA65	Bekaa	Fir	Roots	Collapse, death	FOSC
CA36	Mount Leb.	Hornbeam	Roots, crown	Wilt, dieback, necrotic leaves	FIESC
CA66	Akkar	Cedars	Roots	Collapse, sudden death	FFSC
CA71	Bekaa	Cedars	Roots, crown	Yellowing, decline, death	FOSC
CA68	Bekaa	Cedars	Roots, crown	Yellowing, decline, death	FSSC
CA69	Bekaa	Cedars	Roots, crown	Yellowing, decline, death	FSSC
CA70	Bekaa	Cedars	Roots, crown	Yellowing, decline, death	FSSC
CA72	Bekaa	Pine	Crown area	Yellowing, decline, death	FSSC
CA73	Bekaa	Pine	Crown area	Yellowing, decline, death	FSSC
CA86	Mount Leb	Pine seedlings	Stem, roots	Wilt, death, corky roots	FOSC
CA111	Bekaa	Walnut	Crown area	Crown rot	FSSC
CA8	Bekaa	Cucumber	Roots	Wilting	FFSC
CA117	Mount Leb.	Cucumber	Crown area	Soft rot	FOSC
CA121	South	Melon	Fruits	Black spots, circular blackish lesions	FSAMSC
CA122	South	Melon	Fruits	Black spots, circular blackish lesions	FIESC
CA35	Bekaa	Melon	Roots	Dry rot, browning	FOSC
CA34	Bekaa	Melon	Roots	Dry rot, browning	FSSC
CA50	South	Watermelon	Roots, crown	Wilted and stunted plants, root rot	FOSC
CA51	South	Watermelon	Roots, crown	Wilted and stunted plants, root rot	FOSC
CA23	South	Watermelon	Roots, stem	Wilt, dry rot	FOSC
CA19	Akkar	Citrus	Seedling roots	No symptoms	FIESC
CA16	South	Citrus	Seedling roots	No symptoms	FOSC
CA6	South	Grapefruit	Fruits	Fruit spots, lesions	FTSC
CA4	South	Lemon	Fruits	Fruit spots, lesions	FLSC
CA3	South	Lemon	Stem, twigs	Wilt, dieback, decline	FLSC
CA1	Akkar	Lemon	Stem, twigs	Wilt, dieback, decline	FTSC
CA2	Akkar	Lemon	Stem, twigs	Wilt, dieback, decline	FTSC
CA5	South	Mandarin	Branch	Gummosis, exudates	FTSC
CA59	South	Beans	Collar area	Wilt, rot	FOSC
CA84	Mount Leb	White beans	Roots, crown	Wilt, decline, death	FOSC
CA44	South	Faba beans	Roots	Root rot	FOSC
CA45	North	Peas	Roots, crown	General wilt	FOSC
CA94	Mount Leb.	Peas	Roots	Slow growth	FSSC
CA126	Mount Leb.	Peas	Roots, collar area	Chlorotic dark region on the lower part of the stem	FSSC
CA64	South	Strawberry	Crown area	Dry rot, wilt, death	FFSC
CA63	Mount Leb.	Strawberry	Crown area	Wilt, death	FOSC
CA76	Bekaa	Strawberry	Crown area	Wilt, decline, death	FOSC
CA123	South	Strawberry	Crown area	Wilt, death, dry rot in vascular tissue	FOSC
CA109	North	Strawberry	Roots, crown	Decline, death	FOSC
CA112	Mount Leb.	Ivy	Roots, crown	Decline, death, roots and stem cracking	FSSC
CA89	Beirut	Jacaranda tree	Roots	Wilt, decline, death	FSSC
CA12	Mount Leb.	Poinsettia	Roots	Dieback	FOSC
CA75	Mount Leb.	Poinsettia	Crown area	Wilt, decline, vascular browning	FSSC
CA104	Mount Leb.	Roses	Crown area	Decline, death	FSAMSC
CA91	Bekaa	Onion	Bulb	Internal brown rot	FOSC
CA43	Bekaa	Onion	Bulbs	Rot	FOSC
CA90	Bekaa	Onion	Bulb	Internal brown rot	FSSC
CA83	Bekaa	Onion	Roots, crown	Yellowing, weak root system	FSSC
CA17	South	Olive	Seedling roots	No symptom	FFSC
CA15	South	Olive	Seedling roots	No symptom	FOSC
CA18	South	Olive	Seedling roots	No symptom	FOSC
CA21	South	Olive	Seedling roots	No symptom	FOSC
CA110	Mount Leb.	Apple	Roots	Wilt, death	FSSC
CA48	Mount Leb.	Red apple	Fruits	Fruit rot	FTSC
CA49	Mount Leb.	Red apple	Fruits	Fruit rot	FTSC
CA133	Mount Leb.	Avocado	Roots	Symptoms associated with ambrosia	FSSC
CA87	South	Avocado	Stem bark	Twigs and stem boring	FSSC
CA131	Mount Leb.	Basil	Stem, crown area	Black necrotic lesions on stem	FOSC
CA62	Bekaa	Cauliflower	Roots, crown	Wilt, internal browning	FOSC
CA113	Mount Leb.	Kiwi	Twigs	Decline, dieback	FOSC
CA124	Mount Leb.	Parsley	Roots	Yellowing	FOSC
CA78	Mount Leb.	Parsley	Roots, crown	Wilt, decline, death	FRSC
CA81	Mount Leb.	*Paspalum* grass	Roots	Leaf yellowing, root rot	FIESC
CA80	Mount Leb.	*Paspalum* grass	Roots	Leaf yellowing, root rot	FOSC

**Table 2:** Fusarium species distribution in the different Lebanese regions.

Geographical Origin	*Fusarium* Strains	Frequency of *Fusarium* Species (%)								
		FOSC	FSSC	FSAMSC	FFSC	FTSC	FIESC	FLSC	FRSC	FBSC
South	45	48.9	22.2	2.2	13.3	4.4	2.2	4.4	0	2.2
Bekaa	42	59.5	31	4.8	4.8	0	0	0	0	0
Mount Lebanon	25	52	24	4	0	8.0	8.0	0	4	0
Akkar	17	43.7	12.5	12.5	12.5	12.5	6.3	0	0	0
North	4	100	0	0	0	0	0	0	0	0
Beirut	1	0	100	0	0	0	0	0	0	0

## Data Availability

The data presented in this study are available within the article.
